# Article processing charges, funding, and open access publishing at *Journal of Experimental & Clinical Assisted Reproduction*

**DOI:** 10.1186/1743-1050-2-1

**Published:** 2005-01-13

**Authors:** Eric Scott Sills, Tina Thibault Vincent, Gianpiero D Palermo

**Affiliations:** 1 Division of Reproductive Endocrinology and Infertility, Department of Obstetrics and Gynecology, Atlanta Medical Center, Atlanta, Georgia USA; 2 Journal of Experimental & Clinical Assisted Reproduction, Atlanta, Georgia USA; 3 Cornell Institute for Reproductive Medicine, Weill Medical College of Cornell University, New York, New York USA

## Abstract

Journal of Experimental & Clinical Assisted Reproduction is an Open Access, online, electronic journal published by BioMed Central with full contents available to the scientific and medical community free of charge to all readers. Authors maintain the copyright to their own work, a policy facilitating dissemination of data to the widest possible audience without requiring permission from the publisher. This Open Access publishing model is subsidized by authors (or their institutions/funding agencies) in the form of a single £330 article processing charge (APC), due at the time of manuscript acceptance for publication. Payment of the APC is not a condition for formal peer review and does not apply to articles rejected after review. Additionally, this fee is waived for authors whose institutions are BioMed Central members or where genuine financial hardship exists. Considering ordinary publication fees related to page charges and reprints, the APC at Journal of Experimental & Clinical Assisted Reproduction is comparable to costs associated with publishing in some traditional print journals, and is less expensive than many. Implementation of the APC within this Open Access framework is envisioned as a modern research-friendly policy that supports networking among investigators, brings new research into reach rapidly, and empowers authors with greater control over their own scholarly publications.

## Introduction

*Journal of Experimental & Clinical Assisted Reproduction *is a scientific and clinical journal established in September 2004, offering rapid peer review of research of the advanced reproductive technologies. Content is administered by two chief editors with offices in New York and Atlanta, with peer review supported by an international editorial board. Importantly, all published manuscripts are freely available to a global audience in full text format to facilitate sharing of investigative insights, laboratory methods, and surgical techniques. No other journal presently has these objectives. The journal considers submissions of the following types: Original research, Reviews, Book reviews, Case reports, Commentaries, Debate articles, Hypotheses, Methodology articles and Short reports. All manuscripts published by *Journal of Experimental & Clinical Assisted Reproduction *are included by PubMed at the National Library of Medicine (United States) [1], as well as by the national libraries of the Netherlands [2], Germany [3], and France [4].

### The evolution of modern academic publishing

Creation of the "Open Access" publishing model represented a watershed moment in how research manuscripts are processed, paid for, provided, and protected. Historically, it was the end consumer (the library or reader) who was charged for access to medical, scientific and technical literature. Such payment was traditionally in the form of subscriptions or by access fees levied in exchange for controlled access to particular articles via internet. In contrast, Open Access publishing asks the author to offset the cost of access.

The advent of internet technology facilitated the indexing of medical literature with a speed and degree of sophistication impossible before the world wide web. Nevertheless, once an article was located via computer database search, generally only the 250 word abstract could be viewed – even though full text was often required. As no library can offer each issue of every journal, the literature collection/review process increasingly has become a multi-institutional endeavor. Unfortunately the high cost of maintaining multiple subscriptions has actually caused some libraries to provide fewer journals for users [5,6].

### More to know, but less to read?

With journal holdings at some libraries trending down, smaller academic publishers were early casualties of the reduced demand. Many were lost to consolidation. This resulted in a once diverse publishing community being controlled by only a few organizations with little incentive to change. Meanwhile the number of specialty journals multiplied to keep pace with the growing complexities of medical research. Even as modern research became more multidisciplinary, academic publishers did not make it easy to share findings across dissimilar journals without the reader paying a price. Thus, a paradoxical albeit unintended contraction of access to published medical research resulted. Within the world of academic publishing, limited competition may have registered profits for some but the worldwide impact on readers' access to scholarly literature has been distinctly negative [7].

It was against this "restricted access" background that the Open Access model of academic publishing was conceived, with a view to maximize rapid collaboration among researchers using a relatively new resource with tremendous potential – the world wide web. Open Access publishing incorporates internet technology which frees the investigator from the limitations of a particular institution's journal holdings as well as the delay of the interlibrary loan.

### Open Access and the future of academic publishing

Implementation of the Open Access publishing model has several important benefits for the scientific community. First, published works are available full text via internet thus making them rapidly accessible to a global audience. Since authors maintain the copyright to their own work, they are free to reproduce it on their own website, link it to related sites, or distribute it according to their own needs without obtaining the publisher's permission first. The only requirement is to acknowledge the article's source. "Reprint requests" are obviated since full text manuscripts are available for free via internet. Such networking has already been shown to boost article citations and impact since these manuscripts are easy to find [8,9]. Second, investigators need only an internet connection and a computer to access every published article with open access – no longer are they limited by their library's journal subscription list.

The Open Access publishing model is subsidized by authors (or their institutions/funding agencies) in the form of a single £330 article processing charge (APC), collected at the time of manuscript acceptance for publication. Payment of the APC is never a condition for any manuscript's formal peer review and does not apply to articles rejected after peer review. Additionally, this fee is not assessed for authors whose institutions are BioMed Central members or where genuine financial hardship exists. Such waiver requests are considered by the chief editors on a case-by-case basis. Since the journal exists as an internet resource there is no page limit to the number of color photographs, diagrams, or figures attached to a particular article. Movie files (<10 MB) accompanying a manuscript may also be published using the journal's electronic publishing platform, a feature particularly suited for articles demonstrating new operative or laboratory techniques. Given the full range of publishing possibilities at *Journal of Experimental & Clinical Assisted Reproduction*, it is hoped that the £330 charge will be affirmed as a good value, especially since subsequent costs associated with reprint fees or page charges assessed by many traditional print journals can easily exceed this amount.

## Conclusion

While several journals have moved to offer free internet access to selected articles, this should not be confused with Open Access [10]. Free access is typically associated with a 6–12 month delay between publication and actual availability of a manuscript, and even when full-text articles are provided copyright restrictions limit the reader's distribution and reproduction of the work. In contrast, the APC assessed by Journal of Experimental & Clinical Assisted Reproduction guarantees Open Access to all published work and permits the author to keep the copyright to their own data, thus benefiting all who are interested in advancing research in the assisted reproductive technologies.

## Abbreviations

APC = article-processing charge.

## Competing interests

The author(s) declare that they have no competing interests.
